# Geographic Disparity in Chronic Obstructive Pulmonary Disease (COPD) Mortality Rates among the Taiwan Population

**DOI:** 10.1371/journal.pone.0098170

**Published:** 2014-05-20

**Authors:** Ta-Chien Chan, Po-Huang Chiang, Ming-Daw Su, Hsuan-Wen Wang, Michael Shi-yung Liu

**Affiliations:** 1 Research Center for Humanities and Social Sciences, Academia Sinica, Taipei, Taiwan, Republic of China (R.O.C.); 2 Institute of Population Health Sciences, National Health Research Institutes, Zhunan, Taiwan, Republic of China (R.O.C.); 3 Department of Bioenvironmental Systems Engineering, National Taiwan University, Taipei, Taiwan, Republic of China (R.O.C.); 4 Master of Public Health Program, School of Public Health, National Taiwan University, Taipei, Taiwan, Republic of China (R.O.C.); 5 Division of Family Medicine, Fangliao General Hospital, Pingtung, Taiwan, Republic of China (R.O.C.); 6 Institute of Taiwan History, Academia Sinica, Taipei, Taiwan, Republic of China (R.O.C.); Kliniken der Stadt Köln gGmbH, Germany

## Abstract

Chronic obstructive pulmonary disease (COPD) causes a high disease burden among the elderly worldwide. In Taiwan, the long-term temporal trend of COPD mortality is declining, but the geographical disparity of the disease is not yet known. Nationwide COPD age-adjusted mortality at the township level during 1999–2007 is used for elucidating the geographical distribution of the disease. With an ordinary least squares (OLS) model and geographically weighted regression (GWR), the ecologic risk factors such as smoking rate, area deprivation index, tuberculosis exposure, percentage of aborigines, density of health care facilities, air pollution and altitude are all considered in both models to evaluate their effects on mortality. Global and local Moran’s I are used for examining their spatial autocorrelation and identifying clusters. During the study period, the COPD age-adjusted mortality rates in males declined from 26.83 to 19.67 per 100,000 population, and those in females declined from 8.98 to 5.70 per 100,000 population. Overall, males’ COPD mortality rate was around three times higher than females’. In the results of GWR, the median coefficients of smoking rate, the percentage of aborigines, PM_10_ and the altitude are positively correlated with COPD mortality in males and females. The median value of density of health care facilities is negatively correlated with COPD mortality. The overall adjusted R-squares are about 20% higher in the GWR model than in the OLS model. The local Moran’s I of the GWR’s residuals reflected the consistent high-high cluster in southern Taiwan. The findings indicate that geographical disparities in COPD mortality exist. Future epidemiological investigation is required to understand the specific risk factors within the clustering areas.

## Introduction

According to the global burden of disease reported by the World Health Organization [1], chronic obstructive pulmonary disease (COPD) was the fourth leading cause of death in 2004, and caused 3 million deaths, which corresponded to 5.1% of all deaths globally. The prevalence of COPD worldwide was around 63.6 million cases, and it was also an important leading cause of moderate to severe disability among the elderly. One projection showed that COPD will become the third leading cause of death in 2020 [2]. In Taiwan, the temporal trend of the age-adjusted combined bronchitis, emphysema and asthma mortality rate has been declining sharply, from 33.2 per 100,000 in 1981 to 3.9 per 100,000 in 2007 [3]. However, the specific COPD International Classification of Diseases, Ninth Revision (ICD-9) code 496 was not included in computing COPD-related mortality. Thus, overall COPD-related mortality was greatly underestimated in Taiwan.

The estimated prevalence of moderate to severe COPD among adults aged ≧30 years was 5.4% in Taiwan, while the average prevalence in the 12 Asia-Pacific countries including Taiwan was 6.3%, and in the United States it was 4.8% [4]. With the increasing aging trend for the next few decades, the COPD prevalence among the elderly is expected to surge, and will cause a critical impact on health care and long-term care systems. One economic estimate of the direct medical cost and indirect cost such as lost work and productivity caused by COPD was that it cost a total of $38.8 billion in 2005 in the United States [5]. The higher severity of COPD hospitalized patients resulted in greater medical costs than those for patients with moderate conditions [6]. Thus, combating the threat of COPD will be the top priority of public health. The major risk factors of COPD are smoking [7], exposure to biomass smoke [8], air pollution [9], [10], age [11], gender [12], socio-economic status [13], tuberculosis exposure [14]–[16] and altitude [17]. In addition to these risk factors, spatial clustering and geographic disparities are important information for public health intervention. In the United States, as of 2011 the COPD hospitalization rates were clustered in the eastern and south central areas [18]. In China, the COPD mortality has been shown to be significantly higher among the people living in northern and rural China [19]. Thus, it is important to elucidate the geographical distribution of COPD and figure out possible high risk areas for further epidemiologic investigation and prevention.

In the past, COPD-related studies in Taiwan rarely mentioned the geographical distribution of COPD mortality and possible health disparities. In addition, we will use the complete disease definition of COPD to explore COPD-related mortality. The purpose of this study was to analyze the spatio-temporal clusters of COPD-related mortality and to explore their correlations with the local-specific risk factors.

## Materials and Methods

### Ethics

This study was approved by the institutional review board (IRB) of Academia Sinica (IRB#: AS-IRB-BM 13057). The databases we used were all stripped of identifying information and thus informed consent was not needed.

### Age-adjusted Mortality Rates

The cause of death data at the township level from 1999 to 2007 were collected from the Department of Health in Taiwan, and the mid-year population data were acquired from the Census database of the Ministry of the Interior. The definitions of COPD deaths used the International Classification of Diseases, Ninth Revision (ICD-9) codes 490 (bronchitis, not specified as acute or chronic), 491 (chronic bronchitis), 492 (emphysema), and 496 (chronic airway obstruction, not elsewhere classified). The direct age adjustment method was applied for age-adjusted mortality rates. The reference population was the year-2000 Taiwan population. In addition, direct township-level data on pulmonary tuberculosis incidence and prevalence in the 1990’s were not available. Thus, we used 1994–1999 age-adjusted pulmonary tuberculosis (ICD-9: 011) mortality as a proxy indicator for tuberculosis exposure. The reason why we selected the period 1994–1999 was because the administrative units could be matched with the current spatial units; otherwise the spatial unit might not be consistent for further analysis.

### Smoking Rates

The smoking rates were obtained from the earliest National Health Interview Survey in 2001, which mainly collected information on residents’ health care utilization, expenditure and their health behaviors by a nationwide multistage stratified systematic sampling scheme. Males’ and females’ smoking rates were calculated separately. The spatial unit was the city or county level. Therefore, the heterogeneities within a city or county would be underestimated due to the limitation of the data.

### Air Pollution

The daily air pollution level from 1994 to 1999 was obtained from 73 Environmental Protection Administration (EPA) monitoring stations in Taiwan. The annual average concentration of ambient PM with an aerodynamic diameter ≦10 µm (PM_10,_ µg/m^3^) and the gaseous pollutants SO_2_ (ppb), NO_2_(ppb)_,_ CO(ppm) were computed for each station. Then, we used the empirical Bayesian Kriging [20] in ArcGIS (ArcMap, version10.2; ESRI Inc., Redlands, CA, USA) to interpolate the concentrations throughout Taiwan, and used the zonal statistics function to calculate the average concentration for each township.

### Densities of Health Care Facilities

Because of the privacy issue, we could not get the patients’ exact home address information or which hospitals the patients went to. Thus, we calculated the mean number of the contracted hospitals or clinics in each township from the National Health Insurance Database, which covered nearly 99% of the population in Taiwan [21], as the numerator of the density of health care facilities. To take the size of the different townships into account, the denominator was the size of each township. The density of health care facilities was our proxy for representing the indirect accessibility index.

### Area Deprivation Index and Percentage of Aborigines

The indicators of social economic status (SES) for each township here were area deprivation index and percentage of aborigines. We used population and housing census data in 2000 from the Survey Research Data Archive, Academia Sinica, Taiwan. The area deprivation index was composed of two parts, including the standardized proportion of elementary occupations and the standardized drop-out rate of students aged 15–17 from schooling [22]. The percentages of aborigines were directly computed from the census data.

### Average Altitude in Each Township

The digital elevation data were downloaded from the ASTER GDEM website (http://gdem.ersdac.jspacesystems.or.jp/search.jsp), which is a publicly available dataset for global digital elevation with 30 m * 30 m resolution [23]. Then, we used the zonal statistics function of ArcGIS to calculate the average altitude in each township. The spatial distribution of original digital elevation and the average altitude are shown in Figure S1.

### Statistical Analysis

To understand the relationship between COPD mortality and possible risk factors including smoking, air pollution, density of health care facilities, area deprivation index, pulmonary tuberculosis mortality, the percentage of aborigines and the altitude, we used Geographically Weighted Regression (GWR) to estimate and consider the spatial variability of their relationships [24]. Unlike conventional ordinary least squares (OLS), GWR model is a type of local statistic in which the parameter estimations vary over space. The assumption of spatial non-stationarity is made in GWR, which means the correlations between the independent variable and dependent variables are not the same for every area [25]. In the first step, we apply OLS analysis with SPSS 20.0 (IBM Corp., Armonk, NY, USA) to observe the correlations between COPD mortality and risk factors. Then, we then use free software GWR 4.0 to run the GWR analysis (http://www.st-andrews.ac.uk/geoinformatics/gwr/gwr-downloads/) [26]. The coefficients of each explanatory variable and the R-square in both OLS and GWR will be summarized in the results. The residual maps and the spatial distribution of the significant explanatory variables after GWR will be displayed in the results with ArcGIS (ArcMap, version10.2; ESRI Inc., Redlands, CA, USA). Finally, we use global Moran’s *I,* which is a global test statistic for spatial autocorrelation. The interval of global Moran’s I is between-1 and 1. A higher positive Moran’s I indicates that values in the neighboring areas tend to cluster, while a lower negative Moran’s I implies that higher and lower values are interspersed. When Moran’s I is close to 0, there is no spatial clustering, meaning that the data are randomly distributed [27]. We tested the global spatial autocorrelation of age-adjusted COPD mortality and their residuals after GWR analysis. A Local Moran’s I (LISA) cluster map of the residuals was created for identifying the clusters which cannot be explained by the current risk factors.

## Results

**Figure 1:**
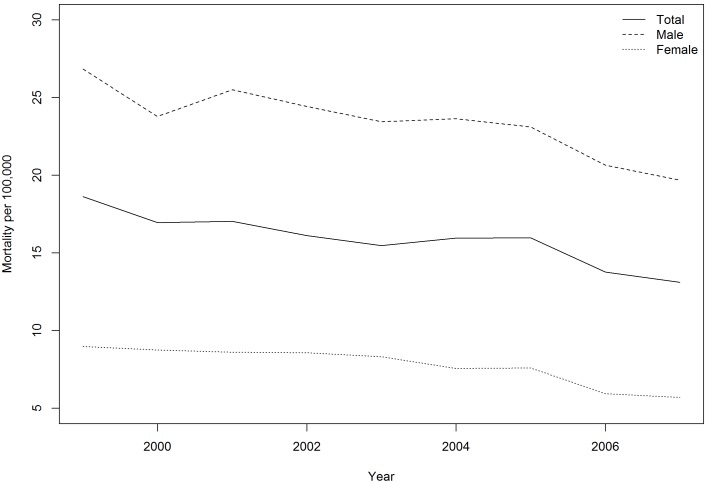
Temporal trend of age-adjusted mortality of chronic obstructive pulmonary disease in Taiwan, 1999–2007.

**Figure 2:**
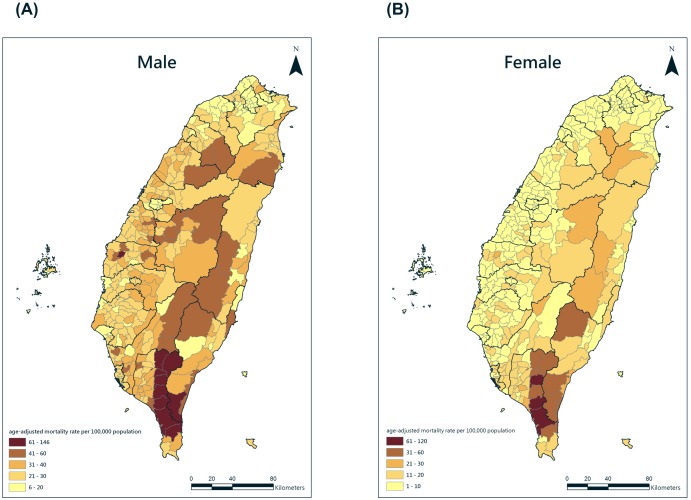
Geographical distribution of age-adjusted mortality of chronic obstructive pulmonary disease in the township level of Taiwan, 1999–2007. (A) Male (B) Female.

During 1999–2007 (Figure 1), the COPD age-adjusted mortality rates for males declined from 26.83 to 19.67 per 100,000 population, and those for females declined from 8.98 to 5.70 per 100,000 population. Overall, males’ COPD mortality rate was around three times higher than females’. In Figure 2, the age-adjusted mortality rates were found to be clustering in southern Taiwan (Males: Moran’s I = 0.22, p<0.05; Females: Moran’s I = 0.19, p<0.05). Although the temporal trend of COPD mortality in Taiwan was going down, geographical disparities still occurred in some townships. Thus, we applied the GWR to identify the effects of risk factors and possible clusters.

In Table 1, the ordinary least square model was applied to model the relationship between COPD age-adjusted mortality and the risk factors. For the males, the male smoking rate, area deprivation index, percentage of male aborigines, CO and PM_10_ all have significant positive correlations with COPD morality (p<0.05). The male tuberculosis mortality, densities of health care facilities, NO_2_ and altitude have significant inverse correlation with COPD mortality (p<0.05). The overall adjusted R-square was 49.4%. For the females, the percentage of female aborigines, CO and PM_10_ all have significant positive correlations with COPD morality (p<0.05). Female smoking rate, female pulmonary tuberculosis mortality, NO_2_ and the altitude have significant inverse correlation with COPD mortality (p<0.05). The overall adjusted R-square was 60.9%. The collinearity was checked by the variance inflation factor (VIF), and the values were all below 7.5, which indicates that there is no collinearity problem.

**Table 1:** The coefficients and R-squares of ordinary least squares model.

Variables	Coefficient	S.E.	p-value	VIF
**Male**				
Male Smoking	40.546	15.291	0.008*	1.529
Area Deprivation Index	0.919	0.465	0.049*	2.367
Male Tuberculosis Mortality	−0.114	0.038	0.003*	2.685
Percentage of Male Aborigines	51.464	4.491	0.000*	3.937
Density of Health Care Facilities	−0.102	0.041	0.014*	1.362
CO	3.126	1.364	0.022*	2.284
NO2	−0.563	0.243	0.021*	3.199
SO2	0.339	0.287	0.238	1.739
PM10	0.438	0.061	0.000*	2.593
Altitude	−0.011	0.003	0.000*	3.296
**Adjusted R-Square**	49.4%			
**Female**				
Female Smoking	−38.877	14.911	0.010*	1.526
Area Deprivation Index	−0.130	0.322	0.686	2.635
Female Tuberculosis Mortality	−0.139	0.047	0.003*	2.364
Percentage of Female Aborigines	46.158	2.938	0.000*	4.318
Density of Health Care Facilities	−0.024	0.026	0.363	1.314
CO	3.376	0.858	0.000*	2.137
NO2	−0.587	0.158	0.000*	3.219
SO2	0.348	0.191	0.069	1.808
PM10	0.281	0.039	0.000*	2.453
Altitude	−0.008	0.002	0.000*	3.036
**Adjusted R-Square**	60.9%			

S.E. = Standard Error, VIF = Variance Inflation Factor.

*p-value <0.05.

In Table 2, the summary statistics of the coefficients for every township are listed. For the males, the medians of all factors’ coefficients are positively correlated with male COPD mortality except for densities of health care facilities and SO_2_. The overall adjusted R-square was 72.2%. For the females, the medians of female smoking rate, percentage of female aborigines, SO_2_, PM_10_ and the altitude were all positively correlated with female COPD mortality. The overall adjusted R-square was 77.9%.

**Table 2:** The coefficients and R-squares of geographically weighted regression.

Variables	N	1^st^ Quartile	Median	3^rd^ Quartile	Robust STD.
**Male**					
Male Smoking	358	−0.512	1.428	2.618	2.320
Area Deprivation Index	358	−0.284	1.288	3.646	2.914
Male Tuberculosis Mortality	358	−1.540	0.195	6.533	5.984
Percentage of Male Aborigines	358	−4.955	2.322	8.017	9.616
Density of Health Care Facilities	358	−2.532	−1.908	−1.128	1.041
CO	358	−0.241	1.952	11.762	8.898
NO2	358	−1.514	1.581	5.041	4.859
SO2	358	−4.956	−0.209	2.117	5.243
PM10	358	−1.327	2.684	5.665	5.183
Altitude	358	−2.276	0.281	2.177	3.301
Adjusted R-Square	72.2%				
**Female**					
Female Smoking	358	−1.748	0.152	1.252	2.224
Area Deprivation Index	358	−2.833	−1.351	0.232	2.272
Female Tuberculosis Mortality	358	−2.686	−1.374	−0.344	1.736
Percentage of Female Aborigines	358	3.584	5.656	12.191	6.380
Density of Health Care Facilities	358	−0.516	−0.287	−0.169	0.257
CO	358	−3.633	−0.492	1.607	3.884
NO2	358	−2.784	−1.014	0.165	2.186
SO2	358	−0.988	0.014	1.731	2.015
PM10	358	1.119	2.385	3.920	2.077
Altitude	358	−1.889	0.834	1.608	2.593
Adjusted R-Square	77.9%				

Robust STD.: Robust standard error (interquartile range/1.349).

**Figure 3:**
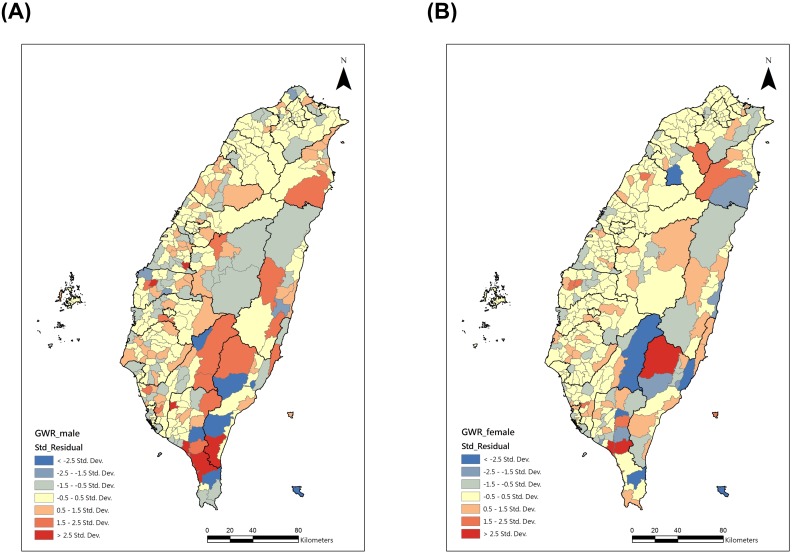
The residual maps after geographically weighted regression. (A) Male (B) Female.

**Figure 4:**
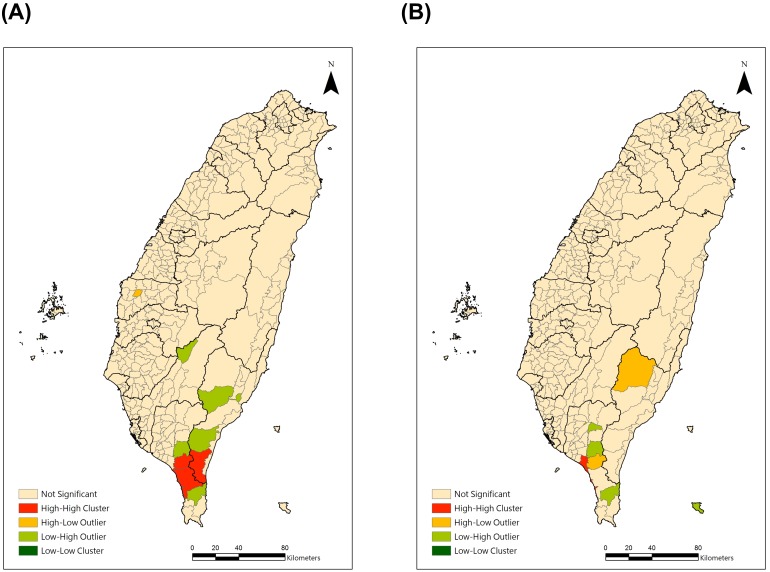
Local Moran’s I of the residuals by geographically weighted regression.

The residual maps after GWR are shown in Figure 3. For both males and females, the spatial autocorrelation of the residuals was not statistically significant, which indicated there was no global spatial clustering after GWR. Global Moran’s I indexes were−0.008 (p = 0.60) for males and −0.007 (p = 0.65) for females, respectively. Furthermore, we used local Moran’s I to find out the local high-high clusters of residuals. For both the males and the females (Figure 4), the high-high clusters were all in southern Taiwan.

**Figure 5:**
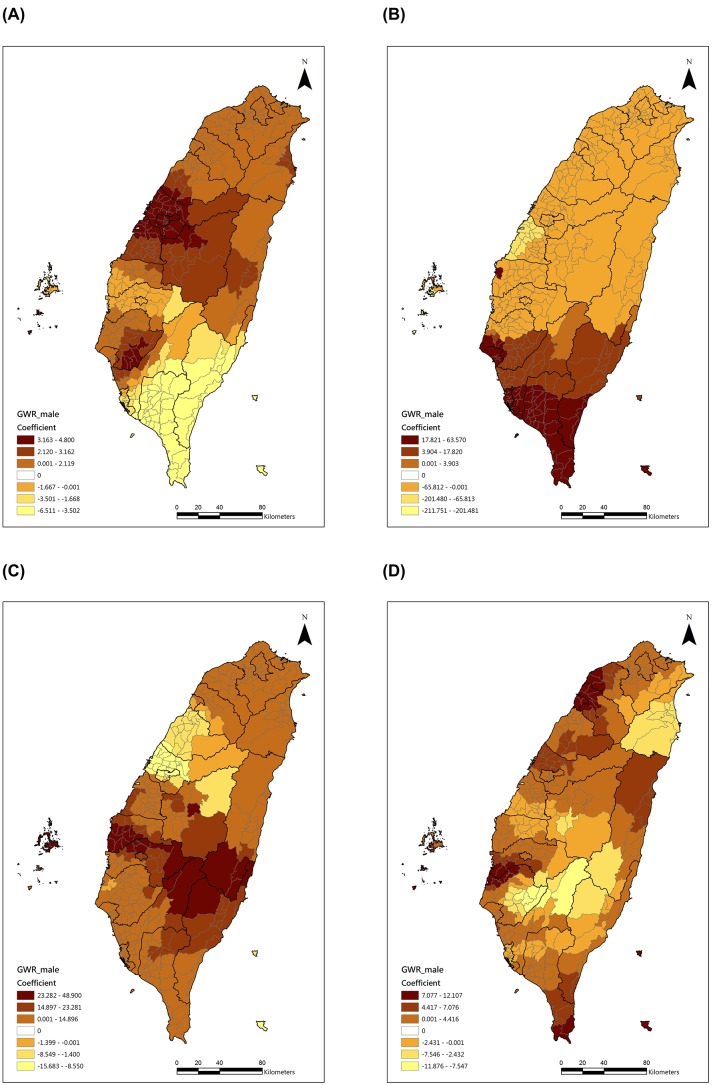
Geographical distributions of the selected significant predictors of male COPD mortality. (A) Male smoking rate, (B) Percentage of male aborigines, (C) CO, (D) PM_10_.

**Figure 6:**
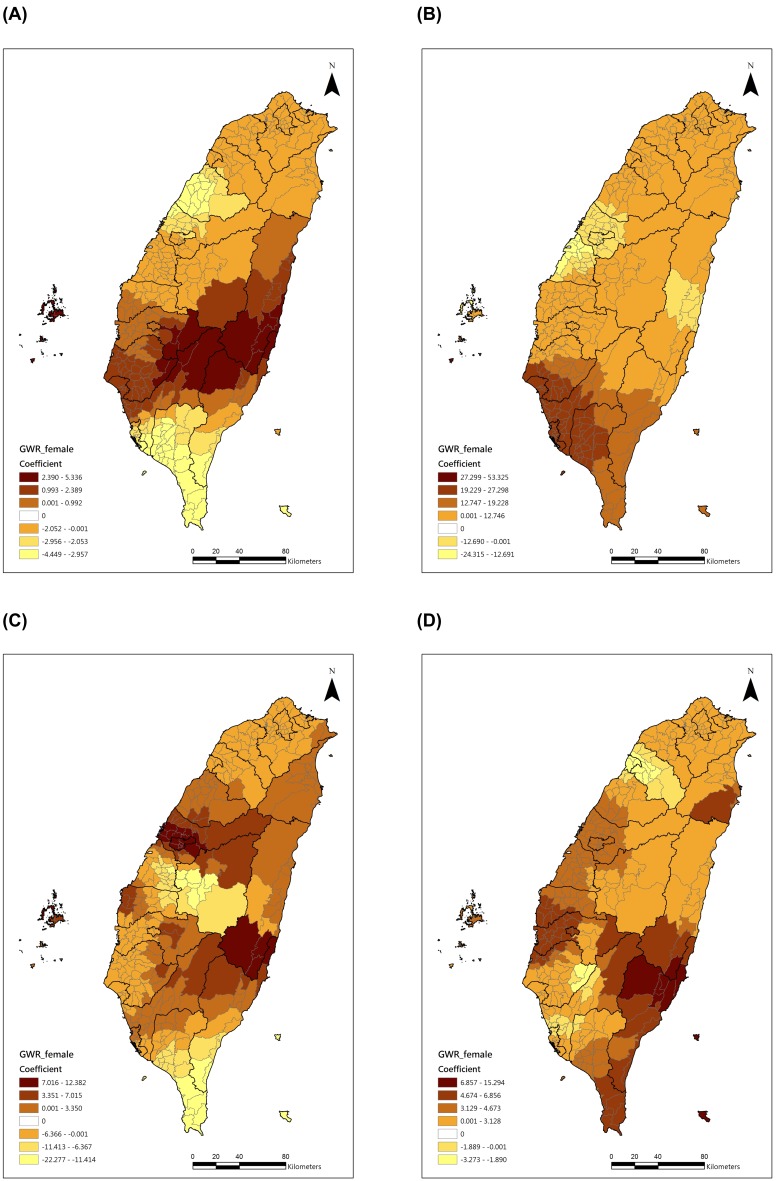
Geographical distributions of the selected significant predictors of female COPD mortality. (A) Female smoking rate, (B) Percentage of female aborigines, (C) CO, (D) PM_10_.

In order to observe the significant risk factors’ effects on the COPD mortality rates, we displayed the selected geographical distribution of the four major risk factors’ coefficients in Figure 5 and Figure 6. For the males (Figure 5), the effect of the male smoking rate was higher in central Taiwan, but the percentage of male aborigines, CO and PM_10_ had higher effects in southern Taiwan. For the females (Figure 6), the female smoking rate had a higher effect in central Taiwan, but the percentage of female aborigines and PM_10_ had higher effects in southern Taiwan.

## Discussion

This paper presents a 9-year spatial-temporal distribution of age-adjusted chronic obstructive pulmonary disease mortality, and identifies possible cluster areas for future epidemiologic investigation. To our knowledge, this is the first paper to address the spatial and health disparity issue of COPD mortality in Taiwan. This study can therefore be a pioneer project laying out the foundation and scheme for broader and longer COPD analysis in Taiwan. The official report of cause of the deaths only reported bronchitis, emphysema and asthma before 2007. The specific diagnosis of chronic airway obstruction (ICD-9: 496) was not included. However, the total number of deaths from chronic airway obstruction during 1999–2007 was 6.8 times more than those from bronchitis (ICD-9: 490), chronic bronchitis (ICD-9: 491) and emphysema (ICD-9: 492). Therefore, we included all these four diagnoses into our disease definition of COPD [28]. The overall temporal trends of COPD mortality have been declining in Taiwan. In the United States, the COPD mortality in males also declined during 2000–2005, but increased in females [29]. The COPD mortality in the Southeast Asia region was estimated at 40.0 per 100,000 population and 79.8 per 100,000 for the Western Pacific region [30]. The average age-adjusted COPD mortality during 1999–2007 in Taiwan was 15.88 per 100,000, which was lower than the average of those two regions. Two possible reasons explaining the declining trend in Taiwan were that the 99% health insurance coverage reduced the economic barrier to health care, and there was a declining trend of smoking rates in the past decade [31].

The conventional OLS model explained total variance of 49.4% for the males and 60.9% for the females. After applying the GWR model, the adjusted R-square increased 22.8% for the males and 17% for females (Males: 72.2%; Females: 77.9%). The global Moran’s I indexes of the residuals were not significant, and the Moran’s I indexes were close to 0, which meant nearly no clustering pattern. With the local Moran’s I, the consistent clusters for both genders were distributed in a few townships of southern Taiwan.

In Figure 3 and Figure 4, the residuals for both genders were high and clustering in southern Taiwan, especially in Pingtung county. There are some possible reasons for these clusters. The most important might be exposure to tuberculosis. Pingtung county had the highest TB incidence (97.5 per 100,000 population) and the second highest TB mortality rate (6.2 per 100,000) in Taiwan in 2011 [32]. It also had the highest TB incidence among the population aged 55–64 (128.0 per 100,000 population) and ≧65 (456.4 per 100,000 population) [32]. However, a longer delay on TB diagnosis and treatment was observed in southern Taiwan [33], which might enhance the risk of getting COPD [15]. Information on the exposure rates to TB in the townships during the 1990’s or before the 1990’s was lacking. Therefore, we used 1994–1999 TB age-adjusted mortality as a proxy indicator. However, an inverse correlation was found between TB mortality and COPD mortality. That might have resulted from the competing risks of death among the elderly [34]. The distribution of age-adjusted pulmonary tuberculosis mortality rates during 1994–1999 is shown in Figure S2.

The effect of the smoking rate was lower in southern Taiwan, which might be underestimated, because the survey results were at the city or county level rather than the township level. The heterogeneity among the townships was unknown, and this was our major limitation. In the southern clusters, nearly 90% of the residents were aborigines, who had a high prevalence of health risk factors such as smoking, drinking and betel nut chewing, causing higher mortality and reducing life expectancy [35].

The socioeconomic status was found to affect the risk of developing COPD and COPD mortality [13]. In our GWR results, the lower densities of health care facilities (both genders), the higher area deprivation index (females), and the higher percentage of aborigines (both genders, Figure S3) were all correlated with an increase in COPD mortality. Although the health insurance coverage reached 99% in Taiwan, access to health care facilities in rural villages was still low. The average socioeconomic status was relatively low among the mountainous aborigines [36]. These factors might hinder proper management of the disease course and result in a worse prognosis. The same pattern was also observed in China, where COPD mortality rates were higher in rural areas than in urban ones (relative risk: 2.14 [1.86–2.46]) [19].

Although ambient air pollution was not conclusively shown to cause COPD [10], the epidemiological data showed that outdoor air pollution had short-term and chronically adverse effects on COPD-related hospital admission and mortality, especially for the elderly [37], [38]. In Taiwan, the higher levels of ambient pollutants increase the risk of hospital admissions [39], [40]. In this study, we found the air pollutants such as CO, PM_10_ and SO_2_ had positive correlations with COPD mortality, and NO_2_ had negative correlations with COPD mortality. However, the highest average concentrations of CO were in northern Taiwan (Figure S4), but these might be correlated with other high concentrations of air pollutants in southern Taiwan, where the clusters of COPD mortality were found.

A previous study in the United States [17] found that COPD mortality had a dose-response correlation with the increasing altitude. The possible biological mechanism was that for persons with respiratory problems, a lower ambient oxygen level might exacerbate hypoxia, pulmonary hypertension and even cause death. In our findings, the OLS results showed a mild inverse correlation between COPD mortality and altitude because most townships (316/358, 88.3%) are located at altitudes less than 500 m. The median values and third quartiles of the GWR estimation in the altitude were positive correlation. This reflects the fact that some townships had positive correlation but some did not. Thus, we further divided the altitudes into four groups (0–500 m, 501–1,000 m, 1001–1500 m, 1501+ m) and compared the COPD mortalities among them (Table S1). It was found that the lowest altitude group had lower COPD mortalities than the other groups. However, there was no clear dose-response relationship due to fewer townships being located at higher altitudes. Therefore, altitude’s effect on COPD mortality was not conclusive in this study.

### Limitations

This study demonstrated some correlations between health behavior, socio-economic status, air pollution and COPD mortality with ecological data. There were some limitations in this study. The first one is lack of a precise smoking rate and tuberculosis prevalence data at the township level and in the 1980’s or 1990’s. These two indicators were declining overall as a temporal trend. Thus, the long-term cumulative effects on COPD mortality rates might be underestimated. In addition, the ambient outdoor air pollution was averaged as one concentration of each air pollutant in each township. The exacerbation of COPD mortality resulting from short-term exposure to elevated concentrations could not be observed from this model. Furthermore, we did not have any information on indoor biomass fuel use, especially from the clustering areas. Therefore, it will be worth conducting further epidemiological studies on those clustering areas to know the specific risk factors locally.

## Conclusion

This study displays the temporal and spatial distribution of COPD mortality in Taiwan. After applying a GWR model and adjusting the known and available risk factors such as smoking, socio-economic status, and air pollution, the spatial variability of COPD mortality in most of the townships can be explained. There are still some abnormal clusters in southern Taiwan. This will need further research to disentangle the local risk factors in order to reduce the disease burdens there.

## Supporting Information

Figure S1 **The spatial distribution of altitude in Taiwan.** (A) Original digital elevation data (30 m*30 m), (B) Average altitude in each township.
(DOCX)

Figure S2 **Pulmonary tuberculosis age-adjusted mortality rates during 1994–1999.** (A) Male, (B) Female.
(DOCX)

Figure S3 **Spatial distribution of the percentage of aboriginal population in Taiwan.** (A) Male, (B) Female.
(DOCX)

Figure S4 **Average concentration of four air pollutants during 1994–1999.** (A) CO, (B) NO2, (C) PM10, (D) SO2.
(DOCX)

Table S1 **Multiple comparison of chronic obstructive pulmonary mortalities among different altitudes.**
(DOCX)

## References

[1] World Health Organization (2008) Part 2: Causes of death. The global burden of disease: 2004 update. Geneva: World Health Organization.

[2] Murray CJ, Lopez AD (1997) Alternative projections of mortality and disability by cause 1990–2020: Global Burden of Disease Study. Lancet 349: 1498–1504. 9167458 10.1016/S0140-6736(96)07492-2

[3] The Ministry of Health and Welfare of the Republic of China (2009) Cause of Deaths. Taipei.

[4] Regional, Copd Working Group (2003) COPD prevalence in 12 Asia-Pacific countries and regions: projections based on the COPD prevalence estimation model. Respirology 8: 192–198. 12753535 10.1046/j.1440-1843.2003.00460.x

[5] Foster TS, Miller JD, Marton JP, Caloyeras JP, Russell MW, et al (2006) Assessment of the economic burden of COPD in the U.S.: a review and synthesis of the literature. COPD 3: 211–218. 17361502 10.1080/15412550601009396

[6] Chiang CH (2008) Cost analysis of chronic obstructive pulmonary disease in a tertiary care setting in Taiwan. Respirology 13: 689–694. 18513247 10.1111/j.1440-1843.2008.01308.x

[7] Forey BA, Thornton AJ, Lee PN (2011) Systematic review with meta-analysis of the epidemiological evidence relating smoking to COPD, chronic bronchitis and emphysema. BMC Pulm Med 11: 36. 21672193 10.1186/1471-2466-11-36 PMC3128042

[8] Hu G, Zhou Y, Tian J, Yao W, Li J, et al (2010) Risk of COPD from exposure to biomass smoke: a metaanalysis. Chest 138: 20–31. 20139228 10.1378/chest.08-2114

[9] Liu Y, Lee K, Perez-Padilla R, Hudson NL, Mannino DM (2008) Outdoor and indoor air pollution and COPD-related diseases in high- and low-income countries. Int J Tuberc Lung Dis 12: 115–127. 18230243

[10] Schikowski T, Mills IC, Anderson HR, Cohen A, Hansell A, et al (2014) Ambient air pollution: a cause of COPD? Eur Respir J 43: 250–263. 23471349 10.1183/09031936.00100112

[11] van Durme YM, Verhamme KM, Stijnen T, van Rooij FJ, Van Pottelberge GR, et al (2009) Prevalence, incidence, and lifetime risk for the development of COPD in the elderly: the Rotterdam study. Chest 135: 368–377. 19201711 10.1378/chest.08-0684

[12] Rycroft CE, Heyes A, Lanza L, Becker K (2012) Epidemiology of chronic obstructive pulmonary disease: a literature review. Int J Chron Obstruct Pulmon Dis 7: 457–494. 22927753 10.2147/COPD.S32330 PMC3422122

[13] Gershon AS, Dolmage TE, Stephenson A, Jackson B (2012) Chronic obstructive pulmonary disease and socioeconomic status: a systematic review. COPD 9: 216–226. 22497534 10.3109/15412555.2011.648030

[14] Chakrabarti B, Calverley PM, Davies PD (2007) Tuberculosis and its incidence, special nature, and relationship with chronic obstructive pulmonary disease. Int J Chron Obstruct Pulmon Dis 2: 263–272. 18229564 PMC2695198

[15] Lee CH, Lee MC, Lin HH, Shu CC, Wang JY, et al (2012) Pulmonary tuberculosis and delay in anti-tuberculous treatment are important risk factors for chronic obstructive pulmonary disease. PLoS One 7: e37978. 22662259 10.1371/journal.pone.0037978 PMC3360660

[16] Sun YC (2013) A dangerous combination: tuberculosis and chronic obstructive pulmonary disease. Chin Med J (Engl) 126: 2203–2204. 23786925

[17] Ezzati M, Horwitz ME, Thomas DS, Friedman AB, Roach R, et al (2012) Altitude, life expectancy and mortality from ischaemic heart disease, stroke, COPD and cancers: national population-based analysis of US counties. J Epidemiol Community Health 66: e17. 21406589 10.1136/jech.2010.112938

[18] Holt JB, Zhang X, Presley-Cantrell L, Croft JB (2011) Geographic disparities in chronic obstructive pulmonary disease (COPD) hospitalization among Medicare beneficiaries in the United States. Int J Chron Obstruct Pulmon Dis 6: 321–328. 21697996 10.2147/COPD.S19945 PMC3119107

[19] Reilly KH, Gu D, Duan X, Wu X, Chen CS, et al (2008) Risk factors for chronic obstructive pulmonary disease mortality in Chinese adults. Am J Epidemiol 167: 998–1004. 18256446 10.1093/aje/kwm393

[20] Pilz J, Spock G (2008) Why do we need and how should we implement Bayesian kriging methods. Stochastic Environmental Research and Risk Assessment 22: 621–632.

[21] Lee YC, Huang YT, Tsai YW, Huang SM, Kuo KN, et al (2010) The impact of universal National Health Insurance on population health: the experience of Taiwan. BMC Health Serv Res 10: 225. 20682077 10.1186/1472-6963-10-225 PMC2924329

[22] Lin HC (2001) Area Deprivation and Mortality in Taiwan. Taipei National Taiwan University. 126 p.

[23] Tachikawa T, Kaku M, Iwasaki A, Gesch D, Michael O, et al.. (2011) ASTER GDEM Version 2 Validation. In: NASA Land Processes Distributed Active, Archive Center and the Joint Japan-US ASTER Science Team, editors. Sioux Falls, South Dakota.

[24] Dijkstra A, Janssen F, De Bakker M, Bos J, Lub R, et al (2013) Using Spatial Analysis to Predict Health Care Use at the Local Level: A Case Study of Type 2 Diabetes Medication Use and Its Association with Demographic Change and Socioeconomic Status. PLoS One 8: e72730. 24023636 10.1371/journal.pone.0072730 PMC3758350

[25] Fotheringham AS, Brunsdon C (1999) Local forms of spatial analysis. Geographical Analysis 31: 340–358.

[26] Nakaya T, Fotheringham AS, Brunsdon C, Charlton M (2005) Geographically weighted Poisson regression for disease association mapping. Stat Med 24: 2695–2717. 16118814 10.1002/sim.2129

[27] Chan TC, King CC (2010) Surveillance and Epidemiology of Infectious Diseases Using Spatial and Temporal Clustering Methods. In: Carlos Castillo-Chavez HC, William B Lober, Mark Thurmond, Daniel Zeng, editor. Infectious Disease Informatics and Biosurveillance: Research, Systems and Case Studies. U.S.A.: Springer. 208–234.

[28] Kuo LC, Yang PC, Kuo SH (2005) Trends in the mortality of chronic obstructive pulmonary disease in Taiwan, 1981–2002. J Formos Med Assoc 104: 89–93. 15765162

[29] Centers for Disease Control and Prevention (2008) Deaths from chronic obstructive pulmonary disease–United States, 2000–2005. MMWR Morb Mortal Wkly Rep 57: 1229–1232. 19008792

[30] Mannino DM, Kiriz VA (2006) Changing the burden of COPD mortality. Int J Chron Obstruct Pulmon Dis 1: 219–233. 18046859 10.2147/copd.2006.1.3.219 PMC2707151

[31] Health Promotion Administration, Ministry of Health and Welfare (2013) The survey on adult smoking behaviors. Taipei Health Promotion Administration, Ministry of Health and Welfare.

[32] Centers for Disease Control, R.O.C. (Taiwan) (2012) Taiwan Tuberculosis Control Report 2012 In: Centers for Disease Control DoH, R.O.C. (Taiwan), editor. Taipei.

[33] Chiang CY, Chang CT, Chang RE, Li CT, Huang RM (2005) Patient and health system delays in the diagnosis and treatment of tuberculosis in Southern Taiwan. Int J Tuberc Lung Dis 9: 1006–1012. 16158893

[34] Berry SD, Ngo L, Samelson EJ, Kiel DP (2010) Competing risk of death: an important consideration in studies of older adults. J Am Geriatr Soc 58: 783–787. 20345862 10.1111/j.1532-5415.2010.02767.x PMC2873048

[35] Wen CP, Tsai SP, Shih YT, Chung WS (2004) Bridging the gap in life expectancy of the aborigines in Taiwan. Int J Epidemiol 33: 320–327. 15082634 10.1093/ije/dyh009

[36] Wang SC, Lee SH, Lee MC, Wang L (2009) The effects of age and aboriginality on the incidence of low birth weight in mountain townships of Taiwan. J Public Health (Oxf) 31: 406–412. 19493914 10.1093/pubmed/fdp052

[37] Bentayeb M, Simoni M, Baiz N, Norback D, Baldacci S, et al (2012) Adverse respiratory effects of outdoor air pollution in the elderly. Int J Tuberc Lung Dis 16: 1149–1161. 22871325 10.5588/ijtld.11.0666

[38] Ko FW, Hui DS (2012) Air pollution and chronic obstructive pulmonary disease. Respirology 17: 395–401. 22142380 10.1111/j.1440-1843.2011.02112.x

[39] Lee IM, Tsai SS, Chang CC, Ho CK, Yang CY (2007) Air pollution and hospital admissions for chronic obstructive pulmonary disease in a tropical city: Kaohsiung, Taiwan. Inhal Toxicol 19: 393–398. 17365044 10.1080/08958370601174818

[40] Yang CY, Chen CJ (2007) Air pollution and hospital admissions for chronic obstructive pulmonary disease in a subtropical city: Taipei, Taiwan. J Toxicol Environ Health A 70: 1214–1219. 17573635 10.1080/15287390701380880

