# Supplementary material for: Geographic Disparity in Chronic Obstructive Pulmonary Disease (COPD) Mortality Rates among the Taiwan Population
Source: PLoS One. 2014 May 20;9(5):e98170. doi: 10.1371/journal.pone.0098170 (PMC4028296; doi:10.1371/journal.pone.0098170)

Figure S1 The spatial distribution of altitude in Taiwan

(A) Original digital elevation data (30m*30m), (B) Average altitude in each township


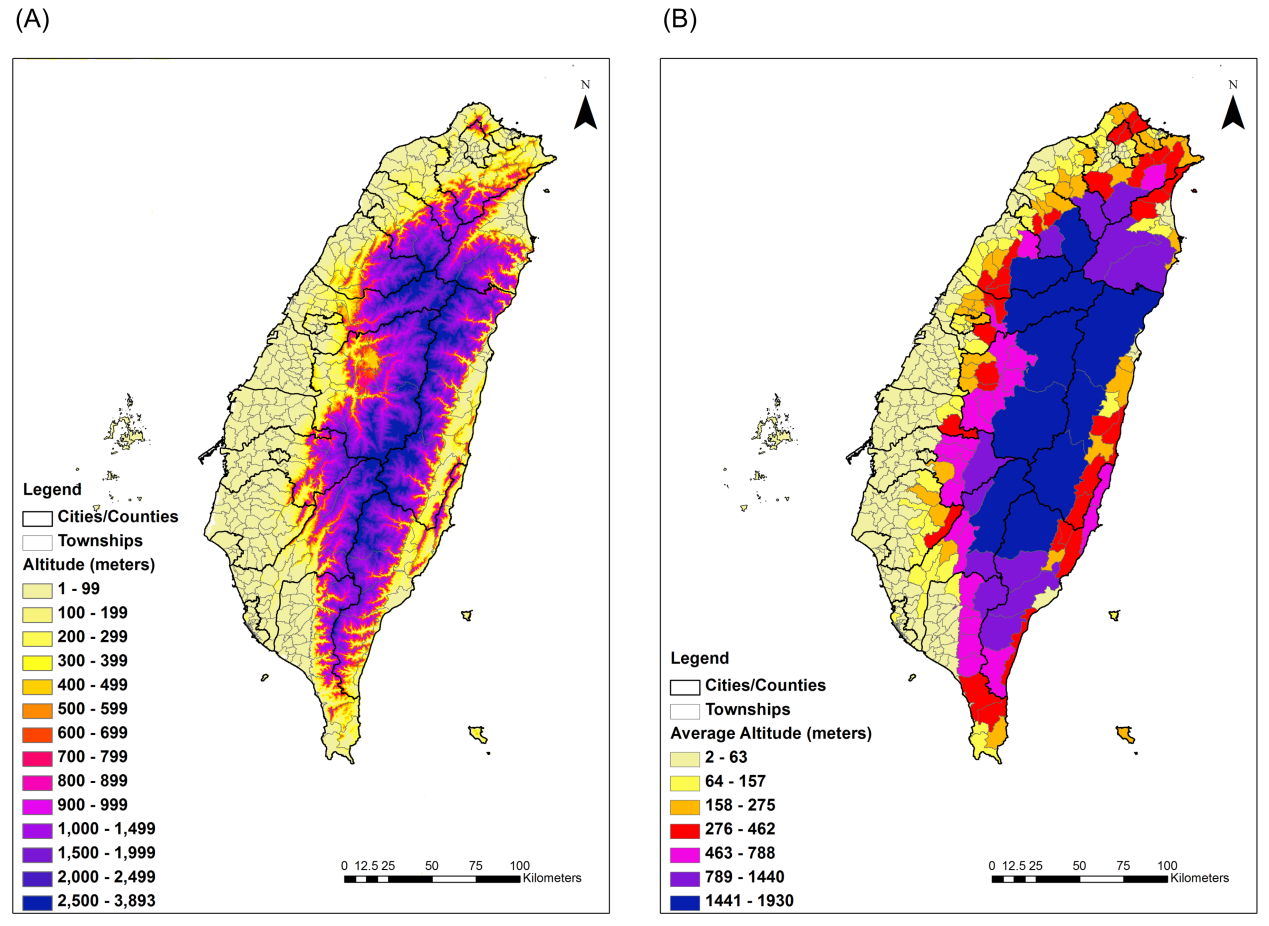

Supplement: Figure S1 — The spatial distribution of altitude in Taiwan. (A) Original digital elevation data (30 m*30 m), (B) Average altitude in each township. (DOCX) [file pone.0098170.s001.docx]
