# Supplementary figures and images for: Geographic Disparity in Chronic Obstructive Pulmonary Disease (COPD) Mortality Rates among the Taiwan Population
Source: PLoS One. 2014 May 20;9(5):e98170. doi: 10.1371/journal.pone.0098170 (PMC4028296; doi:10.1371/journal.pone.0098170)

Figure S2 Pulmonary tuberculosis age-adjusted mortality rates during 1994-1999 (A) Male, (B) Female


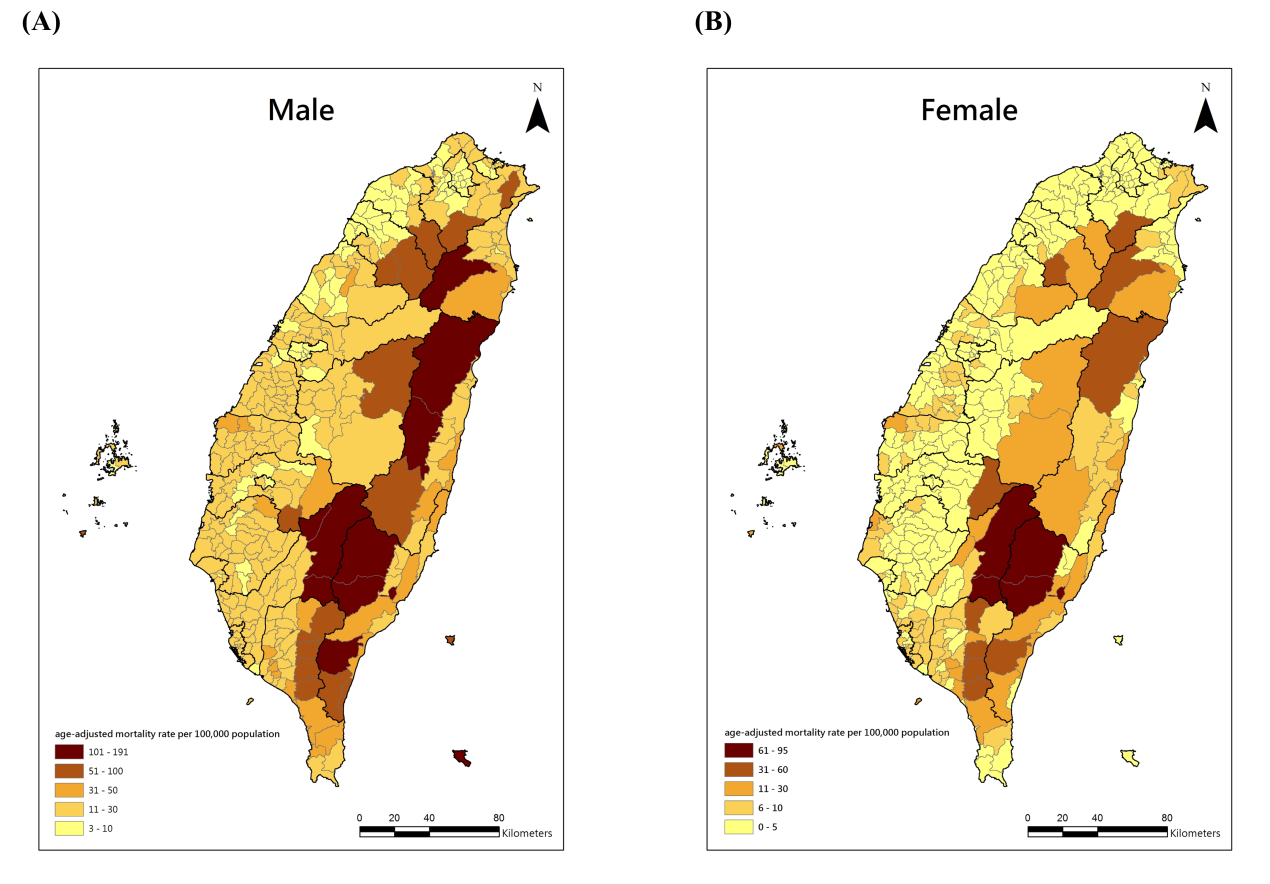

Supplement: Figure S2 — Pulmonary tuberculosis age-adjusted mortality rates during 1994–1999. (A) Male, (B) Female. (DOCX) [file pone.0098170.s002.docx]

**Figure S4 Average concentration of four air pollutants during 1994-1999**

1. **CO, (B) NO2, (C) PM10, (D) SO2**

**
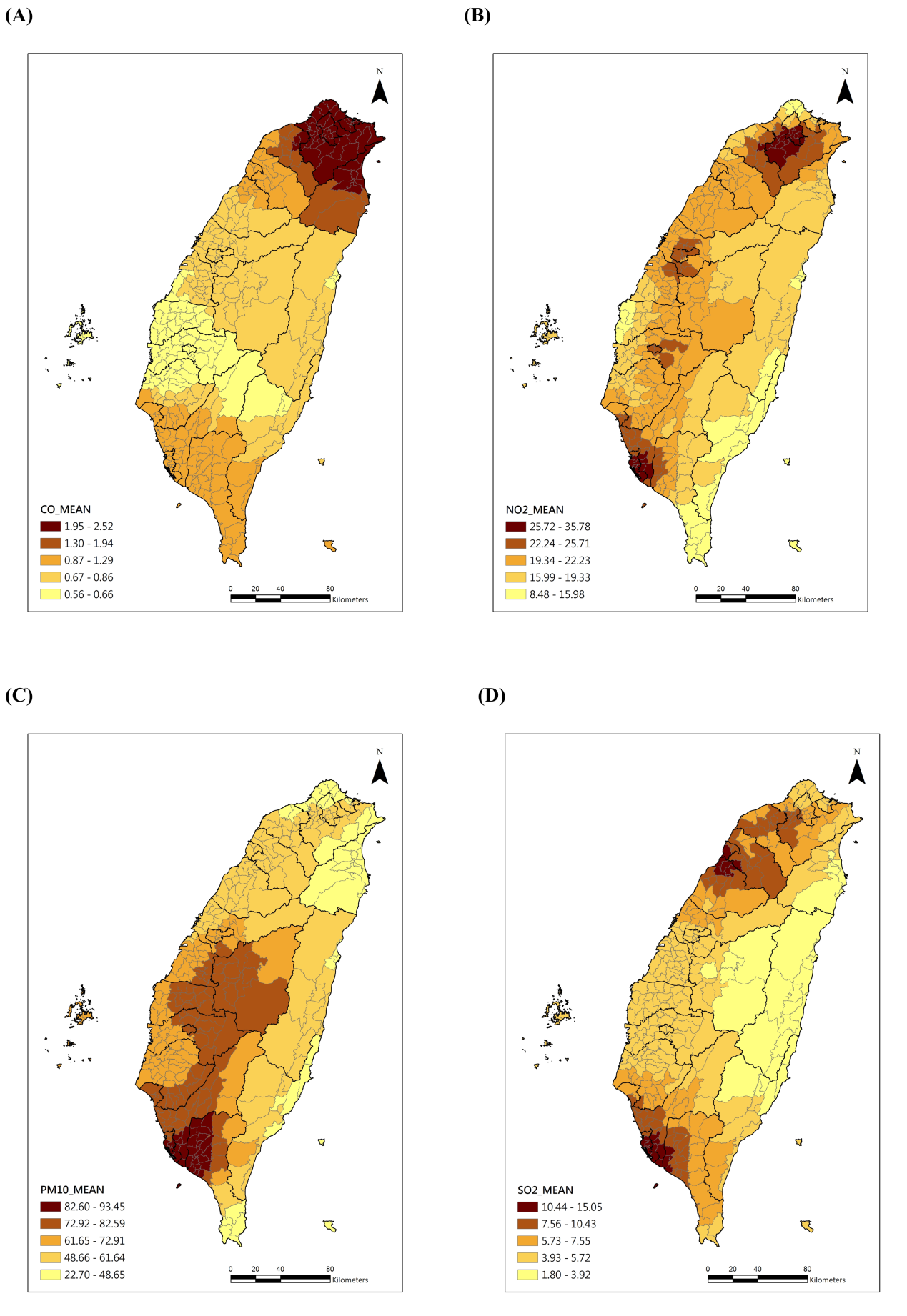
**

Supplement: Figure S4 — Average concentration of four air pollutants during 1994–1999. (A) CO, (B) NO2, (C) PM10, (D) SO2. (DOCX) [file pone.0098170.s004.docx]
