# Supplementary material for: Geographic Disparity in Chronic Obstructive Pulmonary Disease (COPD) Mortality Rates among the Taiwan Population
Source: PLoS One. 2014 May 20;9(5):e98170. doi: 10.1371/journal.pone.0098170 (PMC4028296; doi:10.1371/journal.pone.0098170)
Supplement: Figure S3 — Spatial distribution of the percentage of aboriginal population in Taiwan. (A) Male, (B) Female. (DOCX) [file pone.0098170.s003.docx]

Figure S3 Spatial distribution of the percentage of aboriginal population in Taiwan

(A) Male, (B) Female

**
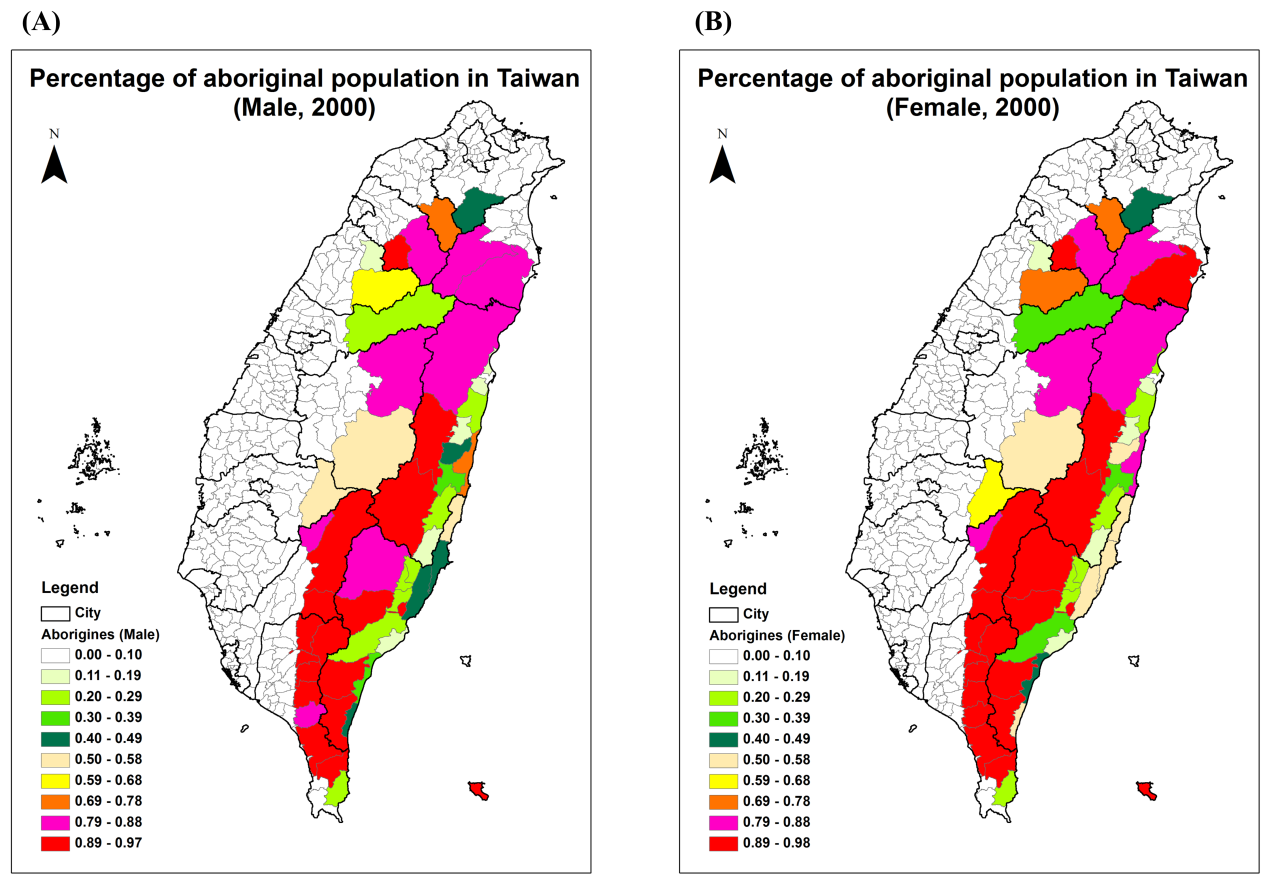
**
