# Supplementary material for: Geographic Disparity in Chronic Obstructive Pulmonary Disease (COPD) Mortality Rates among the Taiwan Population
Source: PLoS One. 2014 May 20;9(5):e98170. doi: 10.1371/journal.pone.0098170 (PMC4028296; doi:10.1371/journal.pone.0098170)
Supplement: Table S1 — Multiple comparison of chronic obstructive pulmonary mortalities among different altitudes. (DOCX) [file pone.0098170.s005.docx]

Table S1 Multiple comparison of chronic obstructive pulmonary mortalities among different altitudes

| Male | | | | |
| --- | --- | --- | --- | --- |
| (I) Altitude (m) | (J) Altitude (m) | Mean Differences (I-J) | Standard Error | P-value |
|  |  |  |  |  |
| 0-500 (N=316) | 501-1000 | -19.599 | 2.837 | 0.000* |
|  | 1001-1500 | -12.723 | 4.441 | 0.027* |
|  | 1501+ | -15.191 | 4.220 | 0.002* |
| 501-1000 (N=23) | 0-500 | 19.599 | 2.837 | 0.000* |
|  | 1001-1500 | 6.876 | 5.165 | 1.000 |
|  | 1501+ | 4.408 | 4.976 | 1.000 |
| 1001-1500 (N=9) | 0-500 | 12.723 | 4.441 | 0.027* |
|  | 501-1000 | -6.876 | 5.165 | 1.000 |
|  | 1501+ | -2.468 | 6.036 | 1.000 |
| 1501+ (N=10) | 0-500 | 15.191 | 4.220 | 0.002* |
|  | 501-1000 | -4.408 | 4.976 | 1.000 |
|  | 1001-1500 | 2.468 | 6.036 | 1.000 |
| Female | | | | |
| 0-500 (N=316) | 501-1000 | -16.310 | 2.037 | 0.000* |
|  | 1001-1500 | -14.570 | 3.188 | 0.000* |
|  | 1501+ | -10.274 | 3.029 | 0.005* |
| 501-1000 (N=23) | 0-500 | 16.310 | 2.037 | 0.000 |
|  | 1001-1500 | 1.739 | 3.708 | 1.000 |
|  | 1501+ | 6.036 | 3.573 | 0.552 |
| 1001-1500 (N=9) | 0-500 | 14.570 | 3.188 | 0.000 |
|  | 501-1000 | -1.739 | 3.708 | 1.000 |
|  | 1501+ | 4.297 | 4.334 | 1.000 |
| 1501+ (N=10) | 0-500 | 10.274 | 3.029 | 0.005* |
|  | 501-1000 | -6.036 | 3.573 | 0.552 |
|  | 1001-1500 | -4.297 | 4.334 | 1.000 |

*p<0.05 with Bonferroni correction
